# Experiments upon Animals

**Published:** 1900-04-14

**Authors:** 


### EXPERIMENTS UPON ANIMALS.

A little time ago many members of the medical profession received a tract issued by some members of the Society of Friends on the subject of experiments on animals, a tract in which serious attention was drawn to what may be called the moral side of the problem—to the question, that is, whether under any circumstances it is justifiable to experiment on animals for the benefit of man. In an able review of the whole subject of the utility and ethics of experiments on animals, which recently appeared in the *British Medical Journal*, the writer states that as the above-mentioned tract was a courteous appeal to the medical profession to turn their minds to the question, and to express their views upon it in the stamped envelopes which were enclosed, he ventured to state the following ethical problem, and to request in answer a plain "Yes" or "No." The problem, abstracted in non-essentials so as to conceal an actual occurrence within the writer's experience, was stated as follows: "A father had a dearly beloved and only child, a grown-up daughter, who fell ill of diphtheria. His physicians told him that without the use of the diphtheria antitoxin she must die. The father asked if the discovery of this antidote was made by experiments on animals, and their answer was to this effect. Again he asked if the supply of the remedy is maintained by operations on animals. The answer was naturally in the affirmative. The father replied that to use the remedy was against his conscience, and that his child's life was in the hands of God. On the premisses of Mr. Coleridge and his friends the father was right; his daughter died. The father now believes that, as every expert knows, by the use of the remedy the life of his child might have been saved, and in his remorse is a broken-hearted man. The question put to Mr. Fry and his co-signatories is, 'Was the father right or was he wrong?' a question which admits of a simple 'Yes' or 'No.' To this question, invited by the Friends' Society, and forwarded to them many weeks ago, no answer has been received." We quote the above because it places in a very striking form the real problem at the bottom of the whole question of experimentation upon animals.

